# Operator-free flow injection analyser

**DOI:** 10.1155/S1463924691000275

**Published:** 1991

**Authors:** Celio Pasquini, Lourival C. de Faria

**Affiliations:** Instituto de Quimica - Universidade Estadual de Campinas C.P. 6154 Campinas SP CEP 13081 Brazil

## Abstract

A flow injection analyser has been constructed to allow an operator-free determination of up to 40 samples. Besides the usual FIA apparatus, the analyser includes a home-made sample introduction device made with three electromechanical three-way valves and an auto-sampler from Technicon which has been adapted to be commanded by an external digital signal. The analyser is controlled by a single board SDK-8085 microcomputer. The necessary interface to couple the analyser components to the microcomputer is also described. The analyser was evaluated for a Cr(VI)-FIA determination showing a very good performance with a relative standard deviation for 15 signals from the injection of 100 μl of a 1.0 mg.ml^-1^ standard Cr(VI) solution being equal to 0.5%.


Journal of Automatic Chemistry, Vol. 13, No. 4 (July–August 1991), pp. 143–146

### Operator-free flow injection analyser

**Celio Pasquini* and Lourival C. de Faria**

*Instituto de Quimica – Universidade Estadual de Campinas C.P. 6154, CEP 13081, Campinas, SP, Brazil*

*A flow injection analyser has been constructed to allow an operator-free determination of up to 40 samples. Besides the usual FIA apparatus, the analyser includes a home-made sample introduction device made with three electromechanical three-way valves and an auto-sampler from Technicon which has been adapted to be commanded by an external digital signal. The analyser is controlled by a single board SDK-8085 microcomputer. The necessary interface to couple the analyser components to the microcomputer is also described. The analyser was evaluated for a Cr(VI)-FIA determination showing a very good performance with a relative standard deviation for 15 signals from the injection of 100 μl of a 1.0 mg.ml$^{-1}$ standard Cr(VI) solution being equal to 0·5%.*

Since its introduction in the middle of the 1970s, flow injection analysis has encouraged a do-it-yourself approach [1]. Most of the advances achieved by the technique in the last decade has resulted from the use of home-made devices constructed to implement new flow manifold configurations and/or to allow the use of a particular chemical reactions [1–6].

The technique is capable of reaching a high degree of automation [7–10]. Although the management of the liquid sample is fully achieved after its introduction to the system, there are other steps that could be implemented to increase the degree of the analyser automation.

It is possible to distinguish four main steps that lead to a fully automated FIA analyser. The first is related to the sample introduction operation. The device employed in this task should be capable of being controlled for a digital signal. The electric energy consume should be low to avoid the use of large DC power suppliers. This requirement agrees with the fact that the device will work in manifolds where the fluid is pumped at relatively low pressure (1–1·5 atm). The device should be capable of introducing sample volumes in the range 10–250 μl.

The second step is related with the automatic sample presentation to the analyser. Expensive autosamplers can be purchased from some dealers. However, old samplers can still be found in many routine laboratories; these were designed to be controlled by motor driven mechanical timers (CAMs). This is particularly true for those laboratories that have been using the air-segmented Auto-Analysers from Technicon.

The other step is the implementation of the detector control and the data acquisition interface. The system is fully automated if data can be stored and processed to report final results and if the output can, in a feedback loop, control at least one major step of the analytical process.

The implementation of only the first two steps will produce an analyser that can perform the mechanical operations required for the analytical process.

This work describes a home-made FIA analyser that is based on an adapted Technicon auto-sampler and on a low-cost sample introduction device. Both can be readily controlled by digital signals from a microcomputer. The resulting instrument can run up to 40 samples or standards without operator intervention.

### Experimental

*The sample introduction device*

Figure 1 shows the sample introduction device. It was built using three electromechanic three-way Teflon valves (NResearch – 161T031, 12 V, 80 mA). The device can be changed from the sampling to the injection mode by turning, simultaneously, all the valves on or off. Therefore, manual operation, if necessary, can be easily implemented.

Injection is achieved when the valves are off, because, for most of the FIA applications, the device can be left in this state most of the time. Furthermore, the sample is not aspirated while the device is in this mode; this results in a substantial reduction in sample usage. Switching on the valves will cause the sample to be aspirated. The aspiration can be provided in many ways. Presently, the instrument employs only gravity by connecting a 50 cm long, 3·0 mm i.d. Tygon tubing to point A in figure 1.

*Auto-sampler adaptation*

An auto-sampler II from Technicon has been used for the sample presentation to the FIA analyser. The instrument was adapted in order to be controlled by an externally

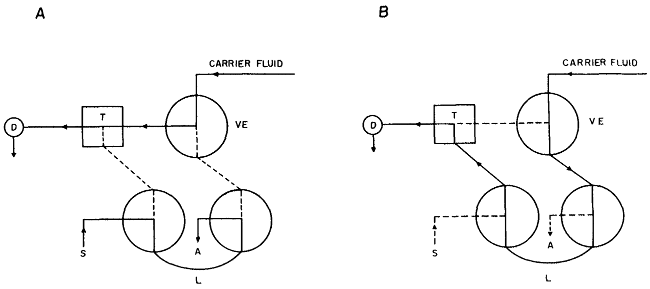

*Figure 1. Sample introduction device diagram. D, detector; T, 't' shaped connector; VE, electromechanical three way valve; S, sample inlet; L, sample loop; A, sample aspiration. A, sampling mode – valves on; B, injection mode – valves off.*

* To whom correspondence should be addressed.

0142–0453/91 $3.00 © 1991 Taylor & Francis Ltd.

generated digital signal. The disk that controlled the time operations in the original sampler has been removed, together with the associated mechanical relay. An electromagnetic relay (12 V coil, 220 V, 2 A) was installed inside the cover of the auto-sampler and the on/off cycle can be now commanded by an external signal. When the electromagnetic relay is on the sampler will retract the sample probe to the washing position and move the tray to the next sample. When the electromechanical relay is switched off the probe will be immersed in the sample. The probe-wash reservoir is not necessary for FIA applications.

The original sample tray can be used. However, a new sample tray was constructed allowing to replace the large sample cuvettes by 2·5 ml, 1·2 cm o.d. sampling glass test-tubes. The new tray was constructed by using an aluminium foil disc (23 cm diameter, 0·5 mm thick). Forty equally spaced holes (1·25 cm diameter) were drilled at the border of the plate. This plate was placed over another plate containing small 0·5 cm holes used to hold the end of the sample tubes. The plates were spaced by acrylic pieces.

*Instrument controller*

The auto-sampler and the sample introduction device were controlled by using a single-board SDK-8085 microcomputer, based on an Intel 8085 CPU. The digital control signals were generated through a 8155 I/O peripheral device. A 74LS373 has been used as a buffer for the 8155 driving signals.

The simple interface, based on TIP 121 and on a BC548 transistors, to a digital TTL control signal is shown as figure 2. Two bits of the port A from the 8155 were used to switch the electromagnetic relay and the valves.

A control program has been written in Assembler for the 8085 following the flow chart in figure 3. The program is as user-friendly as possible. The options are key-stroke selected. For example, pressing the key ⟨F⟩ instructs the software to call the variables program module.

When running, the software allows the operator to program the values of the parameters necessary to the analytical process. The first one is the total number of standards, plus the samples that should be determined. If this parameter is not programmed the controller will not

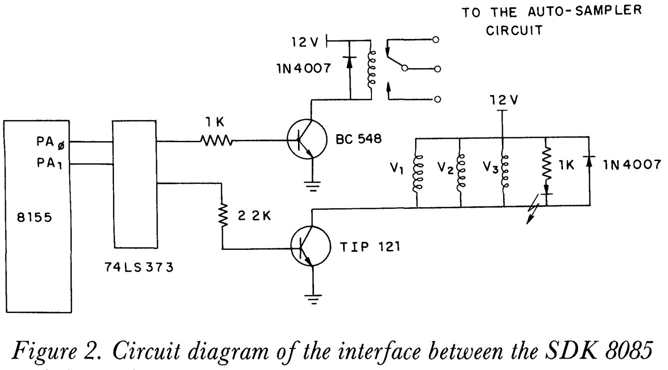

*Figure 2. Circuit diagram of the interface between the SDK 8085 and the analyser components.*

operate the instrument. The other parameters are: the sampling time interval, the injection time interval, the number of replicates for each sample/standard and the extra sampling time interval. Default values are respectively 10, 20 s, triplicate and 0 s. The program checks for out of range values and asks for re-programming if necessary. The time intervals are software generated by calling a subroutine that provides a 1 s delay.

To optimize sample consumption the program will employ a longer sampling time when the sample is accessed for the first time (extra sampling time). The objective is to clean the probe tubing from the previous sample when another sample cuvette is first accessed. Other determinations for that sample, if required, will be carried out employing the user programmed sampling time.

The controller program was recorded in a 2 Kbyte EPROM inserted in the board of the microcomputer, accessed from the initial memory address 3000H. Programmable variables were kept in the 8155 RAM. Copies of the source code, recorded in an IBM compatible format, can be obtained by sending a 5¼ in floppy disk to the authors.

*Other apparatus and FIA manifold*

The fluids were impelled using a ISMATEC MP13 GJ-4 peristaltic pump employing Tygon pumping tubes. The FIA signals were recorded in a Cole-Parmer model 0585 chart recorder. A Micronal model 311 UV/Visible spectrophotometer set at 540 nm has been used to follow the absorbance of the sample/reagent mixture.

The FIA manifold used for Cr(VI) determination is shown as figure 4. Polyethylene, 0·85 mm i.d., tubing was used for building the FIA manifold. A 80 µl, 1 cm optical path Zeiss flow cell was also employed.

All reagents and standards were of analytical grade and were prepared by using fresh deionized water. The Cr(VI) standard solutions were prepared from a 1000 $\mu g.ml^{-1}$ stock solution after suitable dilution.

### Results and discussion

The instrument has been evaluated for a real FIA application. Figure 5 shows results obtained by using the analyser for the Cr(VI) determination, employing the FIA manifold described in figure 3. The relative standard deviation of the height for the 15 peaks found between the two calibration runs is 0·5%. These results show that the sampler device constructed has a performance suitable for FIA applications. The sample volume was changed from 20 to 200 µl, and the absolute precision was kept constant. When using a low sample volume it was necessary to use lower bore sample loop tubing, because the valves arrangement imposes a minimum distance of 4 cm between the sampling loop connection points.

This simple adaptation of an auto-sampler to be commanded by digital signals should be of interest to those

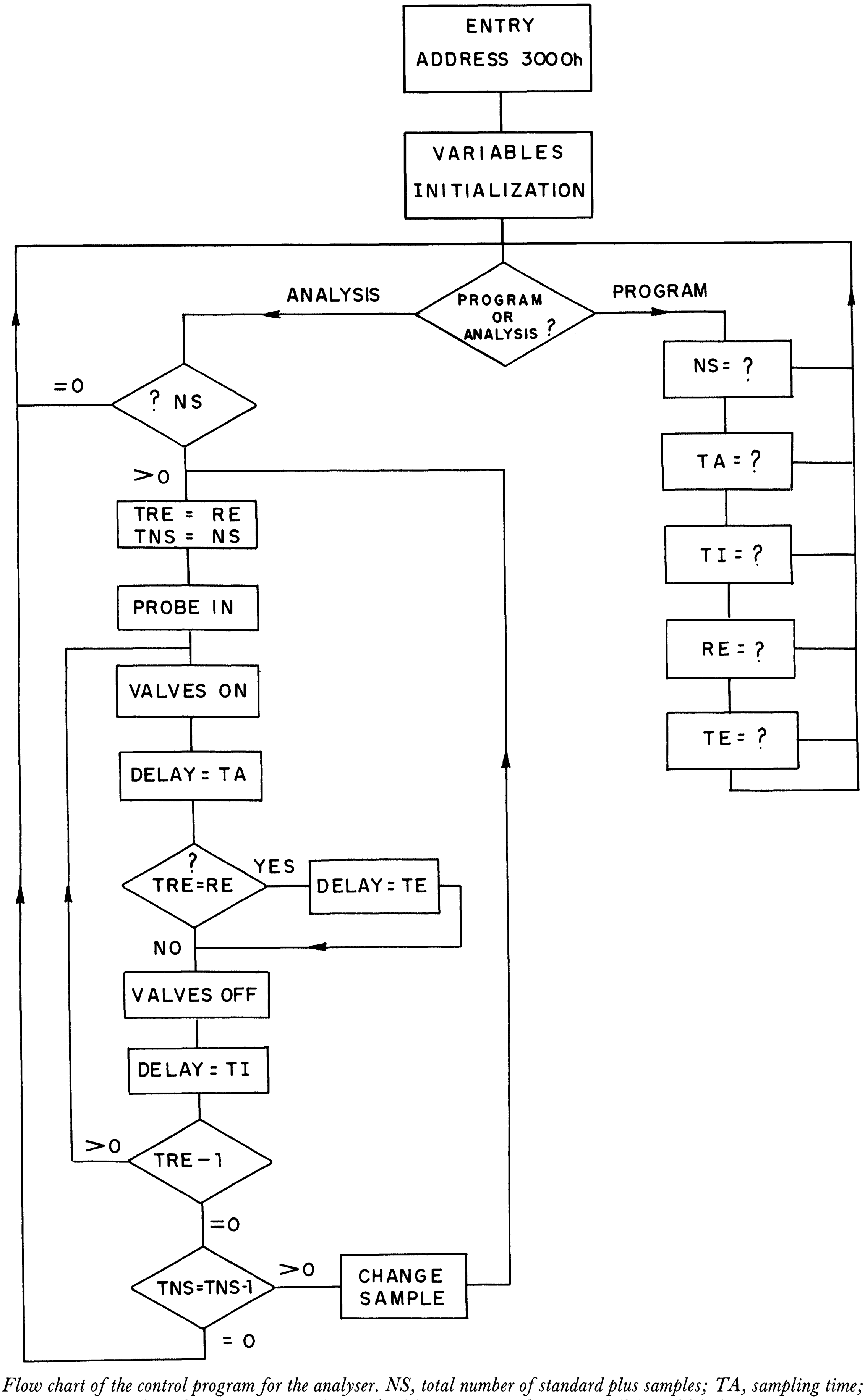

*Figure 3. Flow chart of the control program for the analyser. NS, total number of standard plus samples; TA, sampling time; TI, sample introduction time; RE, number of replicates for each sample; TE, extra sampling time. TRE and TNS, temporary variables initially containing the values of RE and NS, respectively.*

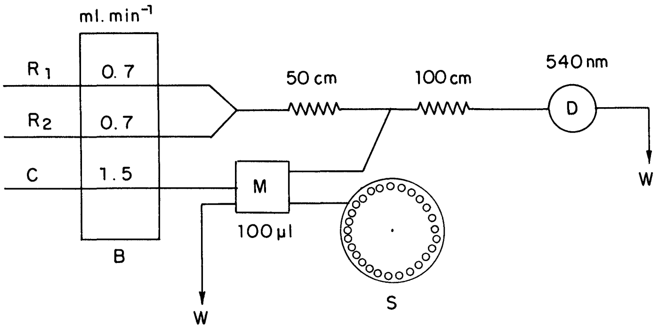

*Figure 4. Flow injection manifold for Cr(VI) spectrophotometric determination. R1, 0·80 M sulphuric acid solution; R2, 0·15% (m/v) diphenylcarbazide solution in 5% acetone; C, water carrier; B, peristaltic pump; M, sample introduction module; S, adapted auto-sampler; D, spectrophotometric detector; W, waste lines.*

laboratories that have been using the air-segmented technique but that have been changing routine work to the FIA methodology.

### Acknowledgement

The authors wish to thank the Laboratory of Clinical Analysis, Hospital of Clinics, UNICAMP, for providing the auto-sampler.

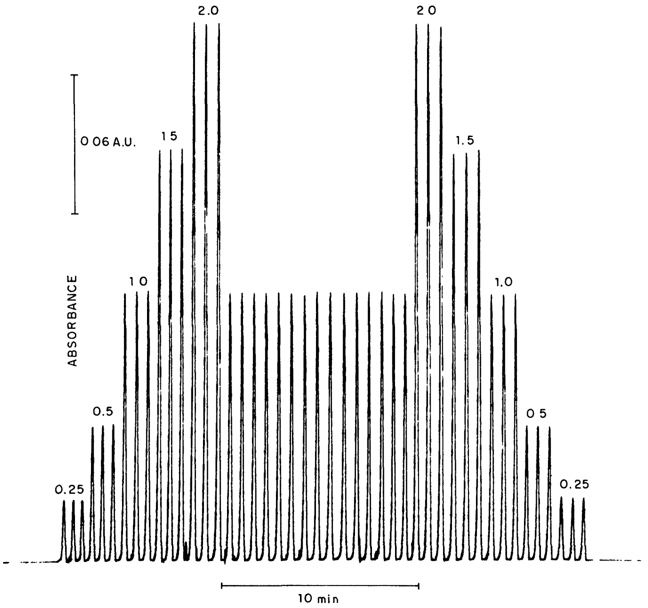

*Figure 5. Results for the reproductibility test for the analyser. The number over the signals are the Cr(VI) standard concentrations in mg.$l^{-1}$. The standards were introduced in triplicate, first in increasing concentration order, followed by 15 introductions of the 1.0 mg.$l^{-1}$ Cr(VI) standard and the standards, again triplicated, in reverse order.*

### References

1. Ruzicka, J. and Hansen, E. H., *Flow Injection Analysis,* 2nd edn (Wiley, New York, 1988).
2. Bergamim Fo., H. Reis, B. F., Jacintho, A. O. and Zagatto, E. A. G., *Analytica Chimica Acta,* **101** (1978), 17.
3. Basson, W. D. and Staden, J. F., *Analyst,* **104** (1979), 419.
4. Pavon, J. L. O., Pinto, C. G., Cordero, B. M. and Mendez, J. H., *Analytical Chemistry,* **62** (1990), 2405.
5. Krug, F. J., Reis, B. F., Gine, M. F. and Zagatto, E. A. G., *Analytica Chimica Acta,* **251** (1983), 39.
6. Reis, B. F., Jacintho, A. O., Mortatti, J., Krug, F. J., Zagatto, E. A. G., Bergamin Fo., H. and Pessenda, L. C. R., *Analytica Chimica Acta,* **123** (1981), 221.
7. Stewart, K. K., Brown, J. F. and Golden, B. M., *Analytica Chimica Acta,* **114** (1980), 119.
8. Prop, L. T. M., Thijssen, P. C. and Dogen, L. G. G., *Talanta,* **32** (1985), 230.
9. Malcome-Lawes, D. J. and Pasquini, C., *Journal of Automatic Chemistry,* **10** (1988), 192.
10. Clark, G. D., Christian, G. D., Ruzicka, J., Anderson, G. H. and Zee, J. A., *Analytical Instrumentation.* **18** (1989), 1.

